# Human Temperaments. XI.—The Man of Action

**Published:** 1916-09-23

**Authors:** 


### HUMAN TEMPERAMENTS.

### XI.—The Man of Action.

The man of action, or of adventurous temperament, abounds in energy which is well under control. It is not the strong, but the weak, who display perpetual fussy movement. The man of action is distinguished in a mixed company by his tranquillity. He has tremendous energy, but he keeps it under control, in reserve, out of sight, ready for action if called upon, but not to be expended without good reason. The quiet, strong man of the popular novelist has incurred a good deal of ridicule, but this is because he has not always been skilfully portrayed, and has sometimes been caricatured. The type exists, and is produced more frequently in England than in most countries.

The man of action is born at all adventure. He springs up as a surprise in the most unexpected quarters. His father may be studious and his mother an invalid, but they both come of good healthy stock. He gets from them a fine, strong, physical constitution, capable of great and long-enduring exertion, slow to feel fatigue, insusceptible to heat and cold, resistant to disease. He can take much exertion with little sleep. He sleeps readily and soundly, but is capable of going long without sleep. His sleep is under control. He can fall asleep when he pleases, wake when he pleases, and take refreshing sleep in snatches when he finds it convenient. He is a fine animal. His senses are keen, and he knows how to use them. His sight is neither long nor short, he has a keen ear, a keen nose, and a sensitive touch, and he is highly observant. He notices things that other people overlook; in particular he is sensitive to shades of expression, and so divines as if by instinct the attitude and feelings of those with whom he is dealing.

Being of boundless and consuming energy, his proclivity is to action, and since his power of inhibition is great, and his energy is under strong control, it is employed not in actuating fine movements of small muscles, but in large, vigorous action of the whole man. Over his finer movements the man of action has complete control. His facial muscles, the muscles of expression, are under the mastery of the will, so that when he pleases he wears an impenetrable mask. He never gesticulates. His superabundant energy does not dribble away in little movements of the fingers and hands. Indeed, his hands and the management of his hands are alone enough to characterise him. They are large and broad and firm. The thumb is well opposed, and even when at rest is on a plane at right angles with his palm. When you shake hands with him, his fingers and metacarpus do not crumple up into a flabby bunch; the hand is as firm as a piece of wood. And it is always in a position to be instantly available. You never catch him with his hands in his pockets. His eye is steady, but it is well opened and alert. It is not attracted by every movement that is going on around him, but nevertheless the movements do not escape him, for he can attend almost equally well to all parts of his field of vision. He is not necessarily very muscular, but he shows in his deep chest the capacity for endurance.

As his energy is great, and is not dissipated in small movements of small muscles, he demands wide spaces to move in. Sedentary life is unbearable to him. He loves the open air and unrestricted freedom of movement. Restriction to the confines of a road is almost as irksome to him as restriction to the confines of a room. He is a man of wide horizons, and the conventions and petty restrictions of the highly organised life of towns press upon him with intolerable restraint, the more so because his consciousness of his own power makes him insensible to fear and contemptuous of danger. He has a well-justified confidence in himself, and longs for the opportunity to put forth the powers that he feels he possesses; and this is the adventurous spirit. Such men are the explorers and adventurers, travellers into strange lands, leaders of romantic expeditions, big-game hunters, mountain-climbers, pioneers, conquerors. Such men were Alexander, Zenghis Khan, Tamerlane, Attila, Pizarro, Cortez, the Cabots, Sir Walter Raleigh, Captain Cook, Sir John Franklin, Marco Polo, Speke, Burton, and many another.

Men of this temperament are fearless. Their control over their energy and bodily powers is so complete that no danger can disturb it. However appalling the position they are in, they remain cool, their faculties alert, their judgment undisturbed. However suddenly the emergency arises, they are not flustered nor perturbed. Nay, it is said of some of the greatest men of action that it required a position of imminent danger to call out their great qualities; that they were never so cool, their judgment never so well balanced and perfect, as in some great emergency. Thus it is with the hunter of big game, who reserves his fire till the charging beast is within a few yards of him; thus it was with the captain of the sailing-ship when the squall struck him unexpectedly; thus it was with Cromwell when his wing was carried away by Rupert's charge; thus it was with Warren Hastings when he was outvoted on his Council, and Nuncomar brought forward his accusation; and thus it was with Clive when his officers mutinied.

It is the same completeness of self-control that gives to the man of action his rapidity of decision. All his powers of body and mind are in hand. They do not need to be summoned, sent for, and collected. They are ready at an instant's notice to be applied in this direction or in that. He seems to jump to his conclusions by an unfailing instinct, but this is very far from his mental process. The alternatives pass through his mind in a flash, and

* The previous articles appeared on May 6, 13, 27, June 10 and 24, July 8 and 22, Aug. 5 and 19, and Sept. 9, 1916.

the long habit of rapid decision enables him to select on the instant.

The man of action is the born leader of men. His strength, his confidence in himself, his complete control over his own powers, give him a similar control over others. In making up his own mind he makes up the minds of others. Mankind may be divided for different purposes in a variety of ways. We may divide them into men of thought and men of action; we may divide them into men of inductive mind and men of deductive mind; we may divide them into those whose passion is for utility and those whose passion is for beauty; we may divide them into the civilised, the semi-civilised, barbarians, savages, and Germans; or we may divide them into leaders and led. Most men belong to the latter class. They have neither the courage, the initiative, the originality, nor the strength of character to strike out a line of conduct for themselves. They are slaves to convention, to fashion, and to custom. They are under the thraldom of what people will think, and no prospect is so terrible to them as that people should think that they are not machine-made and turned out by the gross. Their religion is the religion of the jumping cat, and they dare not move until they see which way the cat is going to jump. Such people without a leader are as sheep having no shepherd. They huddle together, trying to gain confidence from contact with their fellows; and there they will remain, huddled together, without moving this way or that until a leader appears upon the scene; but they are ready, willing, and anxious to follow anyone who inspires them with confidence and commands them with an authoritative voice. They wait for the man of action, and he, with his abounding vitality and self-confidence, can lead them whither he pleases. The confidence that he feels he easily inspires in others, and men instinctively feel and appreciate the quality of leadership. If a dozen men meet for the first time round a table in committee, before half an hour is over one will have established his ascendency, or they will be divided into two groups, each under the dominance of a leader; nay, if half a dozen men get into conversation in a railway carriage, one will have established his ascendency before the end of the journey. When many men are engaged for any time in the same enterprise, they soon fall under the dominance of a leader, even if they begin as a mob, and when once a leader has established his leadership and shown the qualities of the man of action in high degree, there is no sacrifice that his followers will refuse him, no task he can set that they will shrink from. During his life his followers are his slaves, and after his death they deify him.

The men of action leave behind them the greatest names, but they are not the greatest revolutionisers. As often as not their achievements are barren, and prodigious as they loomed in the eyes of contemporaries, they produce little effect on subsequent generations. The history neither of Greece nor of India was much affected by the conquests of Alexander, but the history of Europe has been profoundly affected by Aristotle. Attila might as well have remained at home for all the effect he produced on the history of France. It was Erasmus more than Luther, Voltaire and Rousseau rather than Napoleon, who revolutionised Europe. Bismarck was the greatest man of action of the last half of the nineteenth century, but Bismarck's work is now in a fair way to be undone: the effect of Darwin's work increases year by year. The man of action reaps his reward during his lifetime; the man of thought does not enter into his kingdom till after he is dead. Then

Nations, slowly wise and meanly just,
To buried merit raise the tardy bust.

